# Management of percutaneous treatment of aorta coarctation diagnosed during pregnancy

**DOI:** 10.25122/jml-2021-0363

**Published:** 2022-02

**Authors:** Bogdan Volodymyrovych Cherpak, Yulia Volodymyrivna Davydova, Vitalii Ivanovich Kravchenko, Natalia Sergiivna Yaschuk, Sergii Olegovich Siromakha, Vasil Vasylovych Lazoryshynets

**Affiliations:** 1. Pediatric Cardiology and Cardiosurgery Department, National Amosov Institute of Cardiovascular Surgery NAMS, Kyiv, Ukraine; 2. Obstetrics Department for Extragenital Pathology in Pregnant Women, Institute of Pediatrics, Obstetrics and Gynecology NAMS, Kyiv, Ukraine; 3. Department of Surgical Treatment of Aortic Pathology, National Amosov Institute of Cardiovascular Surgery NAMS, Kyiv, Ukraine; 4. Pediatric Cardiology Intervention Department, National Amosov Institute of Cardiovascular Surgery NAMS, Kyiv, Ukraine; 5. National Amosov Institute of Cardiovascular Surgery NAMS, Kyiv, Ukraine

**Keywords:** coarctation of the aorta, stent implantation, congenital heart diseases, pregnancy, fetus, BP – blood pressure, CHD – congenital heart diseases, CoA – coarctation of the aorta, CP – Cheatham platinum, CT – computer tomography, ECHO – echocardiography, ECS – European Cardiology Society, HTN – hypertension, Mid-aortic S. – mid-aortic syndrome, MRI – magnetic resonance imaging, PDA – patent ductus arteriosus, PG – pressure gradient, ReCoA – aortic coarctation after surgical repair, SBP – systolic blood pressure, SH – systemic hypertension, SI – stent implantation, TTE – transthoracic echocardiography, WHO – World Health Organization

## Abstract

Management of coarctation of the aorta (CoA) during pregnancy is complicated by increased procedural risks to the pregnant woman and her fetus. The aim of this research was to analyze 10-years of experience of CoA treatment diagnosed during pregnancy. During 2010–2020 we performed percutaneous stents implantations (SI) in 4 women during 15–23 weeks of pregnancy and in 6 women 48 hours – 5 years after delivery. In all presented cases, successful CoA repair was achieved. There was a significant decrease of peak-to-peak invasive systolic pressure gradient across the CoA (60.0±31.2 and 11.8±7.3 mmHg, p=0.001) and mean noninvasive systolic arterial pressure (163.0±46.2 and 120.5±9.2 mmHg, p=0.01) after SI. All percutaneously treated women during pregnancy (n=4) delivered healthy full-term babies. At follow-up (from 2 months to 10 years), all 10 women are alive without significant Doppler gradient across CoA with no signs of aortic aneurysm formation. To the best of our knowledge, we presented the largest published cohort of CoA percutaneous treatment during pregnancy. We categorized our experience in managing aortic coarctation diagnosed during pregnancy in one algorithm. Our experience demonstrates that excellent maternal and neonatal pregnancy outcomes can be obtained in women after CoA percutaneous repair, diagnosed during pregnancy. An aortic stent implantation is effective and safe for both mother and fetus.

## Introduction

Coarctation of the aorta (CoA) significantly reduces life expectancy and is associated with increased morbidity even years after successful repair [1]. This congenital heart disease (CHD) is usually diagnosed in childhood, but up to 20% of cases have no clinical manifestations until adult life [2]. The number of women who reach childbearing age with CoA represents most women with CHD during pregnancy [3, 4]. Cardiovascular physiology undergoes profound changes during pregnancy [5]. Stroke volume and heart rate increase from the 3^rd^ to the 7^th^ month of the gravid state, and cardiac output rises by 50% [6, 7]. Blood vessel remodeling occurs due to hormonal factors, which weakens the arterial wall [2, 6]. Pregnancy-related cardiovascular changes place additional stress on coarctation physiology [4, 8, 9]. The blood pressure balance shows sudden changes in the 7^th^ month of pregnancy, despite average blood pressure, being the first critical period for the CoA patient [2]. During labor, blood pressure and cardiac work increase by about 20% at the peak of each uterine contraction, the second critical period [9].

Unrepaired CoA in pregnancy can be life-threatening for both mother and fetus [7]. According to European Cardiology Society (ECS) guidelines for the management of cardiovascular diseases during pregnancy data [10], CoA and recoarctation fall into categories III and IV of the World Health Organization (WHO) classification of maternal cardiovascular risk. Category III lesions are associated with a significant increase in maternal mortality or morbidity and require expert counseling [10]. For pregnant women, complications can include cerebral hemorrhage, aortic dissection, aortic rupture, congestive heart failure, hypertensive crisis, and infective endocarditis [4, 6, 7]. Hypertension is a common consequence of coarctation of the aorta, and treatment in pregnancy is a big challenge [6, 7]. According to ESC recommendation [10], hypertension should be treated, and care should be taken to avoid placental hypoperfusion; however, angiotensin-converting-enzyme inhibitors, angiotensin-receptor blockers, amiodarone, and spironolactone are prohibited. When medical management with typical antihypertensive drugs fails to decrease the coarctation gradient, invasive intervention is indicated [4, 10]. The aim of the current study was to analyze 10-years of experience of CoA treatment diagnosed during pregnancy.

## Material and Methods

From January 2010 to March 2020, we performed 10 CoA percutaneous treatments (mean age 26.4±4.7 years), with CHD diagnosed during pregnancy at the National Institute for Cardiovascular Surgery M.M. Amosov (Kyiv, Ukraine). Stents implantations were performed for 4 women during 15–23 weeks of pregnancy (mean 19.8±3.1 weeks) because of uncontrolled systemic hypertension (SH) with ESC recommended drugs, and 6 women with well-controlled medically SH 48 hours – 5 years after delivery. All patients (n=10) were undergoing obstetric follow-up at the Institute of Pediatrics, Obstetrics and Gynecology Elena M. Lukyanova, National Academy of Medical Science of Ukraine. Arterial systemic hypertension diagnosis and treatment was provided according to ESC guidelines [10]. Preliminary CoA was diagnosed by clinical evaluation and transthoracic echocardiography (TTE). The coarctation anatomy was additionally specified with magnetic resonance imaging (MRI) in 4 pregnant women and computer tomography (CT) in 6 patients. The aortic segments were analyzed and measured angiographically. The statistical analysis was performed using IBM SPSS Statistics 24.0 for Macintosh (IBM Corp. Armonk, NY). Quantitative data are expressed as M±SD (M is the mean, s-standard (mean square) deviation), n is the size of the subgroup analyzed, p is the obtained significance level. The critical significance level was assumed to be 5%.

## Results

All women who had CoA repair during pregnancy (n=4) and 6 patients with postpartum stent implantation had severely arterial hypertension (mean in all group (n=10) systolic – 163.3±49.0 mmHg, diastolic – 87.2±16.8 mmHg). One pregnant woman (20 weeks of gestation) with CoA and controlled hypertension is now under conservative treatment.

The aortic coarctations in our cohort were native in 8 patients, post-surgery in 1 patient (hypoplastic aortic arch in segment B after CoA surgical repair 24 years ago). One pregnant woman was diagnosed with the mid-aortic syndrome, and for one patient, we identified CoA combined with patent ductus arteriosus. Baseline hemodynamic findings of observed women are presented in Table 1. The mean invasive baseline peak-to-peak gradient in our cohort (n=10) was 59.0±29.6 mm Hg. The choice of stent implantation was carried out depending on the type and size of coarctation (Table 1). We applied 3 Cheatham Platinum (CP) (NuMED Canada Inc.) covered stents and 7 bare metals: 1 Palmaz stent PG 3910 (Cordis Corp., Miami, FL, USA), and 6 Andrastents XL (Andramed GmbH, Reutlingen, Germany).

**Table 1.** Baseline hemodynamic findings of observed women (n=10).

**Patient number/pregn. weeks or time after delivery **	**Age (years)**	**Diagnosis**	**S/D blood pressure mmHg**	**PG (TEE), mmHg**	**Invasive PG, mm Hg**	**Type of stent**
**1/19 week (w).**	19	Mid-aortic s	290/110	90	140	CP covered 8Z34
**2/22 w.**	33	CoA	160/80	40	60	Pulmaz 3910
**3/23 w.**	19	CoA with hypoplastic aortic arch	150/80	35	55	CP covered 28 mm
**4/15 w.**	28	ReCoA	160/60	25	50	Andrastent 26XL
**5/2 m.**	29	CoA	130/70	25	45	Andrastent 30XL
**6/48 hr.**	28	CoA+PDA, Ao dissection	150/80	30	55	CP covered 34 mm
**7/6 m**	25	CoA	160/120	25	40	Andrastent 26XL
**8/5 yrs.**	31	CoA	160/80	30	50	Andrastent 30XL
**9/2 yrs.**	22	CoA	140/90	20	35	Andrastent 26XL
**10/1 yr.**	26	CoA	130/75	35	60	Andrastent 30XL

S/D pressure – systolic/diastolic pressure in the left arm (mean in pressure Holter); PG – pressure gradient; Mid-aortic S. – mid-aortic syndrome (suprarenal); CoA – native coarctation of the aorta; ReCoA – aortic coarctation after surgical repair 24 y/ago; Hypoplastic aortic arch (B); CoA+PDA, Ao dissection – Aortic aneurysm dissection type II DeBakey after 24 hours after delivery; PDA – patent ductus arteriosus.

In all presented cases, successful revascularization was achieved. The peak-to-peak systolic pressure gradients (in mmHg) across the coarctation segment decreased significantly (60.0±31.2 and 11.8±7.3, p=0.001) after treatment in all patients (n=10). The mean noninvasive systolic arterial pressure (in mmHg) decreased significantly after stent implantation among the women surveyed (163.0±46.2 and 120.5±9.2, p=0.01).

Complications during stent placement and follow-up were diagnosed in 2 cases: spontaneous rupture of the fetal membrane that was physiologically resolved, and a woman was discharged (pregnancy continued without any complication with normal delivery on term); femoral artery thrombosis that was surgically treated.

All pregnant women treated percutaneously (n=4) delivered healthy full-term babies: 3 by spontaneous uneventful vaginal delivery and 1 (10.0%) by caesarian section (following obstetric indications). Half of the patients undergoing stents implantations (SI) postpartum (n=3) had a caesarian section for different obstetric reasons, and another 3 women – had a spontaneous uneventful vaginal delivery. There was no evidence of any CHD in the newborn in our cohort (n=10), following TTE performed on the fourth day of life. All patients were monitored and discharged 4 to 7 days after treatment. Follow-up studies included clinical evaluations on-demand and scheduled clinical and echocardiographic evaluations at 6 months, 1 year, and every subsequent year. At follow-up (from 2 months to 10 years), all 10 women are alive without significant Doppler gradient across coarctation. A CT scan follow-up was performed in all patients since 2010.

## Discussion

Pregnancy is associated with a 50% increase in plasma volume load, and ventricular function changes and/or valve regurgitation might be expected to progress [11]. Management of CoA during pregnancy is complicated by heightened procedural risks to the pregnant woman and her fetus. For aortic procedures, high estrogen levels impact aortic remodeling, predisposing the aorta to dissection and rupture. To avoid such complications for patients with unrepaired CoA, a CT-scan is recommended every 6–8 months and for those who already had SI – every 2–5 years [3, 4]. However, if the residual arch gradient is low, outcomes of future pregnancies are favorable, with rates of preeclampsia like those of the general population [4]. CoA accounts for around 11% of cases among adults with CHD at our clinic and may be detected for the first time during pregnancy [4, 7], as in our sample (n=10).

The frequency of hypertensive complications during pregnancy in women with CoA in the general population is unknown [12]. The definition of hypertension in pregnancy has changed over time [12, 13]. Older studies included pregnant women with diastolic blood pressure (DBP) ≥90 mm Hg, whereas more recent studies have included women with systolic blood pressure (SBP) ≥140 mm Hg or a DBP ≥90 mm Hg [13, 14]. Similarly, the definition of severe hypertension in pregnancy was reduced from ≥170/110 mm Hg to ≥160/110 mm Hg [14]. In our cohort, severe arterial hypertension was revealed during routine obstetric examination in 5 surveyed women (50.0%). Krieger *et al.* [12] found that the frequency of hypertensive complications of pregnancy had 24.1±3.3% women with CoA *vs.* 8.0±0.1% women without it (multivariate odds ratio (OR) 3.6, 95% confidence interval (CI 2.5 to 5.2). Preexisting hypertension complicating pregnancy (10.2±2.5% *vs.* 1.0%±0.02%, multivariate OR 10.8, 95% CI 5.9 to 19.8) and pregnancy-induced hypertension (13.9±3.0% *vs.* 7.0%±0.1%, multivariate OR 2.1, 95% CI 1.3 to 3.3) were more common in women with CoA [12].

Beauchesne *et al.* [2] found that in 8 women after repair of aortic coarctation with gestational hypertension, 50% had hemodynamically significant residual aortic gradients. The authors concluded that SH during pregnancy in postcoarctectomy patients is related to significant aortic gradients [2]. Vriend *et al.* [6] suggest that pregnancy may “unmask” residual aortic gradients due to the circulatory changes associated with pregnancy, which corresponds to findings in our study. The invasive baseline peak-to-peak gradient varied in our patients’ group from 35 mmHg up to 140 mmHg (mean PG 60.0±31.2 mm Hg).

In our cohort, 30% of women with heart disease use medication to treat cardiovascular diseases during pregnancy [15, 16]. Increased plasma volume, renal clearance, and liver enzyme activity in pregnant women change the pharmacokinetics of these drugs, often resulting in the need for an increased dose. Fetal well-being is a major concern among pregnant women [16]. Thiazides diuretics and angiotensin-converting enzyme inhibitors are contraindicated in pregnancy [10, 17]. The preferred antihypertensive in CoA is labetalol (an alpha-blocker with nonselective beta-blocking properties), which does not affect the uteroplacental blood flow [10, 17]. In our investigation, women (n=11) with diagnosed aortic coarctation during pregnancy received antihypertensive drug therapy according to ECS recommendation [10], but in 4 cases (40.0%), it was refractory, so percutaneous treatment was needed immediately. In 6 cases, arterial hypertension was well controlled by a conservative treatment that allowed us to prolongate SI up to the postpartum period.

Additional aortic wall fragility related to estrogenic impregnation in the postpartum period is a significant issue in aortic coarctation treatment management both for percutaneous interventions and surgical repair [18, 19]. The mortality rate of pregnant women with CoA is no more than 3–4% [7, 20]. To the best of our knowledge, there are no documented cases of death or grave complications following CoA percutaneous stenting during pregnancy. Since 2018 ESC [10] has recommended percutaneous management of recoarctation. In such cases, it is pointed out that using a covered stent is possible during pregnancy but should only be performed for refractory hypertension or maternal or fetal compromise [10].

There are two aortic coarctation percutaneous treatment options: balloon angioplasty and stenting. Post dilatation angiography often disclosed intimal tears and long-term follow-up in some patients who developed saccular or fusiform aortic aneurysms [20]. For these reasons, percutaneous CoA balloon angioplasty should be avoided during pregnancy and has never been reported [20].

In our study, all interventions were performed after the 4^th^ month of pregnancy as ESC recommended. By this time, organogenesis is complete, the fetal thyroid is still inactive, and the uterine volume is still small, so there is a greater distance between the fetus and the chest than in later pregnancy months [10]. The radiation dose of the fetus was kept lower than 50 mGy, according to ESC recommendations during all procedures (n=4) [10]. The potential risks of exposure to ionizing radiation of the fetus depend on the stage of pregnancy and the absorbed dose [10]. The risks are highest during organogenesis and the early fetal period, lower in the 2^nd^ trimester, and lowest in the 3^rd^ trimester [10]. In early pregnancy terms (including pre-implantation at 0–8 days), the high frequency of spontaneous abortion makes it difficult to adequately assess X-ray-induced intrauterine mortality, although it occurs at other stages of gestation at a dose >250 mGy. Radiation-induced abnormalities (typical doses of 100–200 mGy) include growth retardation, intellectual disability, malignancies, and neurological consequences. Periods of greatest vulnerability include growth retardation at 8–56 days, microcephaly at 14–105 days, and intellectual deficits/convulsions/severe mental retardation at 56–105 days. Therefore, X-ray examinations and procedures should be performed after the end of the main organogenesis period (>12 weeks of pregnancy) [10].

The first case of percutaneous CoA treatment during pregnancy in our clinic was performed in 2010. Palmaz stent PG 3910 was used for implantation to a 33 y/o woman at 23 weeks of pregnancy, and native CoA was diagnosed because of severe and uncontrolled SH. The procedure was performed successfully, without any complication; invasive PG scientifically decreased.

We diagnosed mid-aortic syndrome (fibro-muscular dysplasia of an abdominal aortic wall) in a 19-years-old woman at 19 weeks of pregnancy and uncontrolled hypertension (240 per 120 mmHg) [21]. Some sporadic cases of mid-aortic syndrome diagnosed during pregnancy were successfully managed just with medication [22]. Management of the stenting procedure in such cases requires the use of high-pressure balloons, given the extremely high rigidity of the vessel at the point of constriction. In this case, we used CP covered 8Z34 stent mounted on the 14x40 mm balloon-in-balloon catheter in a position related to the markers on the balloon catheters and radiated simultaneously with high-pressure balloon Atlas 14x20 mm (residual gradient – 20.0 mmHg).

One of the most technically difficult coarctation anatomies for percutaneous treatment is hemodynamic aortic atresia. In this case, blood flow through the aortic isthmus ceases completely, and blood supply to the lower part of the body is ensured only through collateral blood flow. We have experience managing aortic coarctation with hypoplastic aortic arch (segment A) and almost hemodynamic atresia in a 19-years-old woman at 23 weeks of pregnancy with invasive peak-to-peak gradient 55.0 mmHg (Table 1, pt. 3). After the procedure with CP Covered 28mm stent mounted on the 14x35 mm BiB catheter, PG was doubled. The procedure was stopped due to the high risk of aortic rupture or dissection. And the implanted stent was redilatated one month after the first intervention.

Given the significant technical progress in performing an open aortic procedure, aortic recoarctation after previous surgery is rare nowadays [23, 24]. Post-surgical recurrent narrowing of the aorta is quite dense in morphological terms, so stenting does not always result in a complete reduction of the gradient at the site of the surgical suture. One of the possible options for the correct treatment strategy is a preliminary balloon angioplasty narrowing to determine the “compliance” of the aortic wall at this location, with subsequent endoprosthesis implantation to prevent “recoil” of the vessel after aortic distension. We have experience of an Andrastent (26XL) implantation to a 28 y/o woman at 15 weeks of pregnancy with CoA surgical correction 24 years ago, presenting significant hypoplastic aortic arch B segment and severe refractory hypertension. PG after the procedure was reduced 10 times up to 5 mmHg.

To our best knowledge, there are only a few cases of CoA percutaneous treatment during pregnancy [4, 7]. Assaidi *et al.* [7] in 2013 reported their experience in transcatheter balloon dilatation and stenting for native aortic coarctation in a 22-year-old pregnant woman with hypertension. No adverse events or recoarctation was observed at 24-month clinical follow-up. In 2020 Ciresi *et al.* [4] presented a case of an 18-year-old primigravid woman at 11 weeks gestation with unrepaired CoA and severe, non-radiating substernal chest pain for 2 days. A Palmaz 3110 stent on an 18-4-mm delivery balloon was implanted to relieve the arch obstruction.

Coarctation-associated aortopathy, long-standing hypertension, and hormonal changes during pregnancy, such as softening the aorta, increase the risk of aortic rupture or dissection [2, 11, 24]. It is known that more than 50% of aortic dissections in women under the age of 40 occur during or after a gravid state [24].

In our series, one woman had acute type II by DeBakey aortic dissection evidenced on the second day after partum. She had controlled SH with SBP<150 mmHg and DBP<80 mmHg during pregnancy. She was not undergoing an MRI as the intervention was not planned during pregnancy. The additional congenital heart defect – PDA was missed by ECHO probably because of juxtaductal CoA.

From our point of view, CoA and PDA combined with the bicuspid aortic valve and slight enlargement of the ascending aorta up to 34 mm contributed to aortic dissection. CP-covered stent was implanted, including PDA, and supracoronary ascending aorta replacement was performed. We decided not to close PDA with an additional device to prevent consuming time and additional risks. If PDA is diagnosed before or during pregnancy, MRI and possible intervention are strongly recommended to avoid major cardiovascular complications. The postoperative period was uneventful, and patients were discharged from the clinic on the second week after admission.

The femoral artery access during a cardiac catheterization may be accompanied by complications such as bleeding, hematoma, arteriovenous fistula, and the most common is thrombosis [25, 26]. In our cohort, femoral artery thrombosis after percutaneous SI was detected in one case. This complication in our clinic is present in around 0.5% of patients undergoing transcatheter cardiac procedures, including neonates. According to ECS [10] data, vaginal delivery in patients with congenital heart disease is recommended with very few exceptions. The ROPAC data show that elective cesarean section carries no maternal benefit and results in earlier delivery and lower birth weight [10, 27]. Vaginal delivery is associated with less blood loss and a lower risk of infection, venous thrombosis, and embolism and should be advised for most women with CHD [10]. In our series of women undergoing CoA percutaneous treatment during pregnancy, only 1 woman (25.0%) had a caesarian section (by obstetric indications), which is comparable with data of other authors [6, 28]. Our results show that women with CoA can have a successful pregnancy, labor, and delivery outcomes. Preconception assessment is essential in understanding anatomy, repairs, and current physiology, all of which can influence risk in pregnancy. With that foundation, a multidisciplinary cardio-obstetric team can predict and prepare for complications that may occur with superimposed hemodynamics.

During the 2 months – 10 years follow-up, all 11 women are alive and do not have recoarctation or any adverse event.

According to ECS [10], Canada’s [13], and NICE [14] guidelines, we categorized our experience in the management of aortic coarctation diagnosed during pregnancy in one algorithm. Until 2018 if a pregnant woman had systolic blood pressure (SBP) ≥160 mm Hg, we prescribed medications recommended by ECS [10]. In cases where SH could be controlled, blood pressure monitoring was canceled, and the woman was observed by an obstetrician-gynecologist. After delivery, echocardiography and CT were performed to clarify the CoA anatomy, and then, after finishing breastfeeding, we performed SI. In cases of refractory SH, MRI was performed during pregnancy to clarify the CoA anatomy, and then SI was performed. Patients with SBP below 160 mm Hg were not observed by the interventional cardiologist. Since 2019, according to the recommendations of NICE [14], the management of patients who, according to clinical examination and echocardiography, were diagnosed with CoA during pregnancy has changed. All pregnant women with CoA, including patients with SBP below 140 mm Hg, were observed by an interventional cardiologist, and patients with SBP 140-≥150 mm Hg received drug therapy approved by the ECS [10]. In cases with controlled SH, blood pressure monitoring continued, but further diagnosis (echocardiography and CT to clarify the anatomy of CoA) was performed after delivery, and after completing breastfeeding, we performed SI. In refractory SH (especially SBP more than 160 mm Hg), MRI was performed during pregnancy to clarify the CoA anatomy, and then SI was performed.

## Conclusion

Our experience demonstrates that excellent maternal and neonatal pregnancy outcomes can be obtained in women after CoA percutaneous repair, diagnosed during pregnancy. An aortic stent implantation is effective and safe for both mother and fetus. Pregnant women with coarctation of the aorta in combination with additional congenital diseases such as PDA bicuspid aortic valve and especially ascending aorta enlargement should have stricter management during pregnancy. They should be considered by a multidisciplinary team as a candidate for coarctation stenting during pregnancy. Strong cooperation of a multidisciplinary cardio-obstetric team allows successful management of pregnancy and delivery for patients with coarctation of the aorta.

## Acknowledgments

### Conflict of interest

The authors declare no conflict of interest.

### Ethical approval

The study was approved by the Ethics Committee of the Scientific Institute National Institute for Cardiovascular Surgery M.M. Amosov (2010-1). The study protocol complied with the regulations and guidelines for Good Clinical Practice in Ukraine.

### Consent to participate

Oral and written informed consent were obtained from the patients before study inclusion.

### Authorship 

CBV contributed to data analysis and wrote the original draft. DYV contributed to data curation. KVI contributed to the methodology. YNS contributed to data collection. SSO contributed to editing the manuscript. LVV contributed to conceptualizing the study.
